# The epidemiology of transient synovitis in Liverpool, UK

**DOI:** 10.1007/s11832-014-0556-5

**Published:** 2014-01-22

**Authors:** William D. Harrison, A. K. Vooght, R. Singhal, C. E. Bruce, D. C. Perry

**Affiliations:** 1 University of Edinburgh, Edinburgh, UK; 2 Mersey Deanery Surgical Training Scheme, Regatta Place, Liverpool, UK; 3 Alder Hey Children’s Hospital, Eaton Road, Liverpool, L12 2AP UK; 4 Warwick Orthopaedics, Warwick Medical School, University of Warwick, Coventry, CV2 2DX UK

**Keywords:** Epidemiology, Transient synovitis, Deprivation, Seasonality

## Abstract

**Background:**

The epidemiology of transient synovitis is poorly understood, and the aetiology is unknown, although a suggestion of a viral association predominates.

**Purpose:**

This population-based study investigated the epidemiology in order to formulate aetiological theories of pathogenesis.

**Patient and methods:**

Cases in Merseyside were identified between 2004 and 2009. Incidence rates were determined and analysed by age, sex, season and region of residence. Socioeconomic deprivation scores were generated using the Index of Multiple Deprivation, allocated by postcode. Poisson confidence intervals were calculated and Poisson regression was used to check for trends.

**Results:**

Two hundred and fifty-nine cases were identified over 5.5 years. The annual incidence was 25.1 (95 % CI 22.1–28.5) per 100,000 0–14 year-olds. Male to female ratio was 3.2:1 (*p* < 0.001). Mean age at presentation was 5.4 years (95 % CI 5.0–5.8), which demonstrated a near-normal distribution. No relationship was identified between seasonality and incidence (*p* = 0.64). A correlation was identified with socioeconomic deprivation in Merseyside: incidence rate ratio 1.16 (95 % CI 1.06–1.26, *p* < 0.001), although further analysis within the subregion of Liverpool did not confirm this finding (*p* = 0.35).

**Conclusions:**

The normal distribution for age at disease presentation suggests a specific disease entity. The absence of seasonality casts some doubt on the popular theory of a viral aetiology. The absence of a consistent socioeconomic gradient in both Merseyside and Liverpool challenges a previous suggestion of an association with Perthes’ disease. This paper provides ecological evidence that may challenge existing aetiological theories, though transient synovitis remains an enigma.

## Introduction

Transient synovitis (TS) is a self-limiting synovial inflammation of the hip that occurs in childhood. Despite being one of the most common disorders of the paediatric musculoskeletal system [3], very little is known of this disease. The epidemiology and aetiology are poorly understood, with little attention given to its understanding.

The primary clinical focus of TS has been its differentiation from septic arthritis (SA), an orthopaedic emergency that can cause joint destruction and even death. Both present with an atraumatic limp and a varying degree of systemic illness (e.g. fever). The research agenda has mirrored the clinical dilemma, with the development of numerous clinical prediction algorithms to help differentiate TS and SA [2, 7, 12, 21, 22]. However, little attention has been given to the aetiology of transient synovitis.

The epidemiological understanding of TS almost invariably arises from small case series, often with unclear case definitions, and unknown denominator populations [8, 9, 24]. Confusion also arises as the terms “irritable hip” and “transient synovitis” are often used interchangeably, with little consideration given to a more formal diagnostic differentiation. The term “irritable hip” is best used as an umbrella term to describe the clinical presentation of a patient whose hip is irritable, therefore representing a range of diagnoses causing pain or a limp. Transient synovitis pertains to a pathology causing synovial inflammation and an effusion, the diagnosis of which is only reached when all other explanations for an irritable hip have been excluded. These difficulties have limited the epidemiological understanding of TS to date.

Uncertainty has led to various hypotheses to explain the aetiopathogenesis of the condition. The common notion of a viral aetiology is based on five primary research papers, of which three measured immunological parameters and two investigated clinical evidence of a preceding viral illness [3, 5, 10, 11, 23]. Tolat et al. [23] identified that interferon levels were raised in 28 of 65 children with TS, but they did not use a control group. Similar observations were made by Leibowitz et al. [10] in an unadjusted analysis using a control group formed from individuals of all ages. However, whilst this provides some evidence to support a viral hypothesis, the finding of raised interferon levels is not universal, not specific to a virus, and is not made in comparison with a well-formed control group. Additionally, searches for the viral pathogen have failed to produce a consistent result, with a range of viruses isolated in a variable number of children with TS, a finding which is perhaps consistent with any group of infants [10]. Clinical enquiries of viral illness are also inconclusive, with some suggesting a higher rate of preceding gastroenteritis or upper respiratory tract infection [5], though these studies are similarly inconsistent and flawed by recall bias. Perhaps the strongest evidence to support the viral association is the ecological evidence of an association between TS and seasonality [9], a phenomenon that is well established in childhood disease [16]. Evidence of seasonality lacks external corroboration, and other epidemiological features that are known to be associated with viral illness (i.e. socioeconomic deprivation [1]) have not been explored. Another association commonly made in the literature is that of Perthes’ disease, though there is similarly a paucity of evidence to support or refute this.

The study described in the present paper used a defined geographic population group to describe the descriptive epidemiology of TS, with emphasis placed on ascertaining the degree to which ecological patterns support suggested aetiological associations.

## Methods

This was a descriptive observational study of all children presenting to a large tertiary hospital with transient synovitis between January 2004 and September 2009. The protocol in place within the hospital at that time was that any child presenting with hip pain or an atraumatic limp underwent an ultrasound scan of the hips. The cohort was initially established to devise a clinical prediction algorithm to differentiate TS and SA, which has been published previously [21].

Children were included if they were under the age of 14 years at the time of presentation and had documented evidence of a joint effusion on ultrasound examination of the hips. Exclusions were made if an alternative diagnosis later became apparent to explain the symptomatology; this was assessed by reviewing the written and computerised medical notes of each child, with exclusions made on subsequent diagnoses of juvenile idiopathic arthritis, Perthes’ disease, stress fractures and tumours around the hip (*n* = 55). Children undergoing arthrotomy for suspected septic arthritis were similarly excluded (*n* = 42), among whom 29 were believed to truly have septic arthritis and 13 were believed to have transient synovitis. Given the low number of negative arthrotomies (i.e. for transient synovitis), all children undergoing arthrotomy were excluded to ensure that the cohort was homogeneous, therefore minimising false-positive diagnoses of transient synovitis. A diagnosis of transient synovitis was therefore made only for those children with a positive ultrasound scan, spontaneous resolution of symptoms and no subsequent diagnosis that may explain the initial symptomatology.

From those children included, a geographic cohort was formed to identify children who arose from the areas for which the hospital is the major provider of secondary paediatric care (i.e. eliminating children from further afield who may represent more complex tertiary referrals). To define this cohort, postcodes were identified at diagnosis, and the region attributed using an online spatial matching tool (GeoConvert, Mimas, University of Manchester). Study individuals were initially restricted to those within a geographic boundary within the Northwest England metropolitan county of Merseyside (which encompasses individuals within the Liverpool, Sefton and Knowsley local authorities combined). The boundary of Liverpool, a subregion of Merseyside, was examined independently. Alder Hey Children’s Hospital is the sole children’s emergency department within Liverpool, and the major provider of paediatric care for Merseyside, and therefore this geographic restriction ensured that the majority of children attending secondary care services would be captured by this sampling frame. Children with recurrent TS were included once, at their initial presentation, and excluded thereafter. For the purpose of the analysis it was assumed that the population of 0–14-year-old children in the regions remained the same over the study period, based on the child population at the 2001 census.

Deprivation was assessed using the Index of Multiple Deprivation (IMD). The IMD is a national deprivation score allotted by Lower Layer Super Output Areas (LSOA). LSOAs are the lowest layer of UK administrative geography and were geographically defined in 2004 by UKBORDERS (http://www.edina.ac.uk/ukborders). The IMD score and rank were determined for each individual using the postcode via GeoConvert (Mimas, University of Manchester). The number of LSOAs within the study area were equally divided into five levels of deprivation, termed “deprivation quintiles”. Census data were obtained through an online UK census support service (CasWeb, Economic and Social Research Council) to establish the child population for each LSOA quintile. Incidence rates for deprivation quintiles were then calculated. In short, there are 34,378 LSOAs in England and Wales. Each LSOA is scored and ranked according to routine census data sources, which consider (1) population income, (2) employment, (3) health and disability, (4) education, (5) skills and training, (6) barriers to housing and services, (7) living environment and crime. LSOAs represent a minimum population of 1,000 residents and a maximum of 3,000. On average there are 400 households or 1,500 residents within a given LSOA.

### Analysis

The incidence of transient synovitis was analysed by age at presentation, gender and deprivation quintile. Poisson confidence intervals were established, and trends examined with Poisson regression. Seasons were nominally defined as winter (November–January), spring (February–April), summer (May–July) and autumn (August–October). The chi-squared goodness of fit test (*χ*^2^) was used to examine the seasonal distribution, along with additional graphical exploration to ensure the appropriate allocation of season.

The age at presentation was compared to a normal distribution using a Q-Norm plot, in order to identify deviation from the normal distribution. Stata 10.0 (StataCorp, College Station, TX, USA) was used for the analysis.

## Results

### Patients

**Fig. 1:**
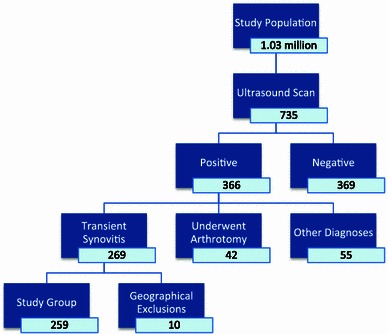
Flow diagram of patient group allocation (*number* indicates the number of patients in each group)

Two hundred and sixty-nine children fulfilled the case definition of TS over the study period (5 years and 9 months). A further 10 were excluded as they arose from outside the geographically defined population boundaries. Hence, the final analysis included 259 children (Fig. 1).

Case ascertainment was over a period of 5 years and 9 months. The total population of children aged 0–14 years old at the 2001 census was 179,397. The total denominator population was therefore 1.03 million child years at risk over the 5¾-year study period. The annual incidence of TS was therefore 25.1 (95 % CI 22.1–28.5) per 100,000 0–14 year olds.

There was a strong preponderance of transient synovitis amongst males, with a 3.2:1 male: female ratio (197 males, 62 females; *χ*^2^*p* < 0.001). No difference was observed in the laterality of disease (118 left hips, 133 right hips, 8 both hips; *χ*^2^*p* = 0.65).

### Deprivation

There was a strong association between the incidence of TS and quintile of deprivation within Merseyside (Table 1). Poisson regression revealed that each unit increase in the deprivation quintile increased the risk of TS by a factor of 1.16 (95 % CI 1.06–1.26) (*p* < 0.001). However, this trend was not observed looking at the Liverpool subregion in isolation (Table 2, *p* = 0.35).

**Table 1:** Incidence of transient synovitis by Index of Multiple Deprivation quintile, Merseyside

Quintile	Population	Cases	Incidence	95 % confidence
	(0–14 years at 2001 Census)			
(Most deprived) 5	40,633	65	27.8	21.5–35.5
4	38,044	70	32.0	24.9–40.4
3	34,059	53	27.1	20.3–35.4
2	33,739	45	23.2	16.9–30.1
(Least deprived) 1	32,922	26	13.7	9.0–20.1

**Table 2:** Poisson confidence intervals for the incidence rates of TS within the deprivation quintiles for the Liverpool local authority

Quintile	Population	Cases	Incidence	95 % confidence
	(0–14 years at 2001 Census)			
(Most deprived) 5	19,750	27	23.8	15.7–34.6
4	19,103	35	31.9	21.2–44.3
3	16,857	27	27.9	18.4–40.5
2	15,733	37	40.9	28.8–56.4
(Least deprived) 1	16,662	26	27.1	17.7–39.8

### Incidence by age at onset

**Fig. 2:**
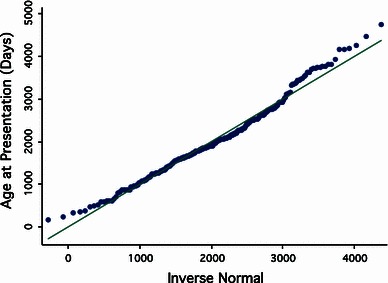
A Q-Norm plot for age at presentation, which demonstrates how closely the data resembles a perfectly normal distribution

The mean age at presentation was 5.4 years (95 % CI 5.0–5.8 years). The youngest age at presentation was 6 months (178 days) and the oldest was 13 years (4,742 days). The data appeared to approximate a normal distribution, though there was a subtle positive skew, evident as an upwards deflection on the Q-Norm plot (Fig. 2). Assuming normality, 95 % of the children diagnosed with TS were between 11 months of age and 10 years 4 months of age.

**Table 3:** Incidence of transient synovitis by season

Season	Cases	Incidence	95 % confidence
Winter	67	26.0	20.1–33.0
Spring	63	24.4	18.8–31.3
Summer	67	26.0	20.1–33.0
Autumn	60	23.3	17.8–30.0

### Seasonal presentation

No significant relationship was identified between the seasonality of TS presentation and the incidence of TS (*χ*^2^ test, *p* = 0.93; Table 3).

**Fig. 3:**
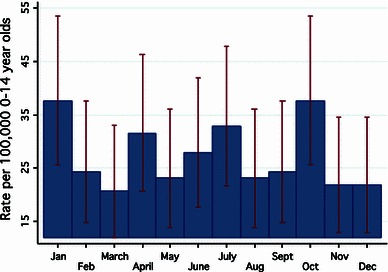
The incidence of TS according to the month at presentation. The bars are Poisson confidence intervals

To ensure that there was no misclassification of season, the monthly presentation of TS was also explored graphically. This similarly confirmed the absence of an association, with significant overlap of error bars (Fig. 3).

## Discussion

The study reported in the present paper explored the epidemiology of TS in a defined population within Merseyside, using a well-defined case definition. These results are of interest to a broad audience and offer some insight into the epidemiology and aetiology of this very common but poorly understood disease.

The incidence of transient synovitis in this study (25.1 per 100,000 person-years) was lower than previous estimates of 76.2–110 per 100,000 person-years [8, 24]. This may be explained by the narrow case definition and the uncertainty over the number of patients with TS that reach secondary care services, given the possibility that significant numbers are exclusively managed in the community. The incidence is therefore a minimal incidence. We acknowledge that this places limitations on the generalisability of results; nevertheless, pragmatic epidemiological research in TS is likely to be either sensitive, with a broad case definition but poor specificity (such as when using general practice diagnosis codes as the case definition [8, 24]), or specific (i.e. ultrasound positive) but with more limited sensitivity, such as in this study. This study utilises a robust population and case definition to make inferences about the aetiology of the disease, acknowledging the potential limitations on the diagnostic sensitivity in this pragmatic approach.

Studies that have investigated the age distribution of the “irritable hip” have failed to demonstrate a normal age distribution [13], but those using a more robust diagnostic definition of TS have obtained better approximations to a normal distribution [8, 9]. We suggest that our finding of a near-normal distribution confirms that the diagnosis made was from a relatively homogeneous cohort of children, therefore suggesting that TS is a single disease entity, rather than a conglomerate of diagnoses (as is encountered when using the term “irritable hip”).

Although the youngest child recorded with TS was 168 days (6 months) old, children <11 months (314 days) old were statistical outliers. As such, caution is required when making a diagnosis of TS in these outlying age groups. Other diagnoses should be considered in these instances, such as septic arthritis or non-accidental injury.

The strong male predominance (3.2:1) and the peak age of disease (5.5 years) share epidemiological similarities to Perthes’ disease of the hip [19]. Examining the deprivation trend across Merseyside appeared to strengthen this association, as a strong deprivation gradient is known in Perthes’ disease [4, 6, 14, 19]. However, further analysis within the Liverpool subregion alone failed to confirm this association. Perthes’ disease is known to demonstrate a strong deprivation gradient in both Liverpool and Merseyside [4, 19, 20]. The age distributions of Perthes’ disease and TS also differ, with Perthes’ demonstrating a lognormal distribution (a very clear positive skew) and TS a normal distribution. It is unclear why the hip of a 5-year-old boy should be so vulnerable to both diseases, but there does not appear to be a clear epidemiological link between the two diseases.

The commonly cited aetiological determinant of TS is a viral aetiology, which is largely based on previous evidence of a seasonal pattern of disease [8]. We were unable to replicate an association with seasonality; nor were we able to identify any trend by month of presentation. In general, the propensity for viral illness varies by age [15], with a mean of 6.0 respiratory viral illnesses per year amongst 1 year olds, 3.6 amongst 5–9 year olds and 2.7 per year in >10 year olds [16, 17]. If TS has a viral aetiology we could expect a peak to occur in early infancy, and therefore TS to be most common in younger children, though this is not the case. The male predominance of TS similarly does not support a viral aetiology. Biochemical research in children with TS has failed to demonstrate a clear correlation with viral illness [10, 11, 24], and whilst studies measuring interferon levels offer some support for a viral association [5, 10], small clinical studies which enquire into recent viral illness are inconsistent and flawed by recall bias.

The association with deprivation in Merseyside is a novel finding. However, this association may be a referral bias, whereby the more disadvantaged individuals attend secondary care services or present to inner-city secondary care services. The absence of a similar association within the Liverpool subregion may support this bias, or may be due to too little variation in deprivation in Liverpool to identify a difference. However, the absence of variation was not apparent when similar methods were applied to examine the epidemiology of Perthes’ disease [18]. Attempts to replicate this in other settings would be useful.

Assigning deprivation by postcode introduces the assumption of homogeneity amongst individuals within each postcode area. Such area measures of deprivation introduce inherent error into the study, although the low number of households within an English LLSOA should limit this error. IMD by postcode is widely used and accepted throughout healthcare, education and government, but individual measures of deprivation undoubtedly supersede area measures.

Ethnicity is a variable that is not addressed in this study. Merseyside is largely composed of the white British ethnic group (86.2 %), with other groups being white other 2.6 %, mixed 2.5 %, Asian 4.2 %, black 2.6 % and Arab 1.2 % [18]. Whilst ethnicity was not recorded, its inclusion was unlikely to have yielded interesting results owing to the absence of ethnic divergence within the population.

Future work should seek to resolve the presence of an association with deprivation, and develop the descriptive epidemiology in order to guide further analytical work. Given the annual incidence, the cumulative (lifetime) incidence of TS within this study is 1:265 children, which may be sufficiently frequent to consider investigating the aetiology using established birth cohorts, allowing a prospective evaluation of risk factors. However, such studies must be mindful of the difficulties involved in assigning a case definition, balancing sensitivity and specificity in the diagnosis. Multicentre studies would be particularly well placed to rapidly resolve questions surrounding the viral aetiology of the disease. Of particular interest would be synovial fluid analysis, analysis of serum immunological markers, and perfusion MRI, all of which may help to elicit the mechanism and determinants of TS.

This descriptive study used a defined case definition of TS to explore the epidemiology, thereby making ecological observations pertaining to the current aetiological theories. Unfortunately, this study creates more questions than it provides answers to, by challenging many of the commonly held beliefs of TS aetiology. TS remains a common but perplexing disease of childhood.
